# Anti-Atherosclerotic Properties of Wild Rice in Low-Density Lipoprotein Receptor Knockout Mice: The Gut Microbiome, Cytokines, and Metabolomics Study

**DOI:** 10.3390/nu11122894

**Published:** 2019-11-28

**Authors:** Mohammed H. Moghadasian, Ramandeep Kaur, Kayla Kostal, Akhila A. Joshi, Mahboubeh Molaei, Khuong Le, Gabor Fischer, Francesca Bonomini, Gaia Favero, Rita Rezzani, Branden S. J. Gregorchuk, Vanessa Leung-Shing, Michelle Wuzinski, Andy I. Seo, Denice C. Bay

**Affiliations:** 1 Department of Human Nutritional Sciences, University of Manitoba, Winnipeg, MB R3T 2N2, Canada; kaurr27@myumanitoba.ca (R.K.); kkostal@sbrc.ca (K.K.); joshiaa@myumanitoba.ca (A.A.J.); 2 Canadian Center for Agri-food Research in Health and Medicine, St. Boniface Hospital Research Center, Winnipeg, MB R2H 2A6, Canada; mahbobe.molaei@gmail.com (M.M.); kle@sbrc.ca (K.L.); 3 Department of Pathology, University of Manitoba, Winnipeg, MB R3T 2N2, Canada; Gabor.Fischer@umanitoba.ca; 4 Department of Clinical and Experimental Sciences, University of Brescia, 25121 Brescia, Italy; francesca.bonomini@unibs.it (F.B.); gaia.favero@unibs.it (G.F.); rita.rezzani@unibs.it (R.R.); 5 Department of Medical Microbiology & Infectious Diseases, University of Manitoba, Winnipeg, MB R3E 0J9, Canadadenice.bay@umanitoba.ca (D.C.B.)

**Keywords:** wild rice, microbiome, metabolomics, atherosclerosis, LDL-r-KO mice, cytokines, 16S rDNA, plasma, feces, proteins, carbohydrates, functional food

## Abstract

Background and aim: We previously reported the anti-atherogenic properties of wild rice in low-density lipoprotein receptor knockout (LDL-r-KO) mice. The present study aimed to discover the mechanism of action for such effects. Materials: Fecal and plasma samples from the wild rice treated and control mice were used. Fecal bacterial population was estimated while using 16S rDNA technology. The plasma samples were used to estimate the levels of 35 inflammatory markers and metabolomics, while using Meso Scale multiplex assay and liquid chromatography-mass spectrometry (LC-MS/MS) techniques. Results: Many bacteria, particularly *Anaeroplasma sp*., *Acetatifactor sp*., and *Prophyromonadaceae sp*., were found in higher quantities in the feces of wild rice fed mice as compared to the controls. Cytokine profiles were significantly different between the plasma of treated and control mice. Among them, an increase in the level of IL-10 and erythropoietin (EPO) could explain the anti-atherogenic properties of wild rice. Among many metabolites tested in plasma of these animals, surprisingly, we found an approximately 60% increase in the levels of glucose in the wild rice fed mice as compared to that in the control mice. Conclusion: Additional studies warrant further investigation of the interplay among gut microbiome, inflammatory status, and macronutrient metabolism.

## 1. Introduction

Appropriate types of diets and levels of physical activities are believed to be major determinants of maintaining optimal health [1,2]. Many studies have reported that regular consumption of certain foods, particularly plant-based foods, such as whole grains, fruits, and vegetables, as well as fish, are associated with decreased prevalence of chronic diseases, specifically cardiovascular disease [3,4]. Phytochemicals that are contained within these foods are believed to mediate these health benefits and include phytosterols, dietary fiber, dietary antioxidants, oleic acid, and docosahexaenoic acid (DHA). On the other hand, food ingredients, such as saturated fat, heavy metals, and other contaminants, may increase the risk of cardiovascular disease [5,6]. One of the common chronic diseases with a significant negative impact on the quality of life is atherosclerotic vascular disease, which remains the main cause of global morbidity and mortality [7]. A fundamental contributor in the pathogenesis of atherosclerosis is the oxidation of low-density lipoprotein (LDL) particles, which are taken up by macrophages, initiating foam cell formation in the arterial wall [8]. Therefore, foods with an ability to lower LDL cholesterol and prevent LDL oxidation have been at the center of atherosclerosis prevention [3].

Wild rice has many health benefits when consumed, as noted in historical documents of the indigenous peoples of North America for centuries, as well as other nations, including Chinese and Europeans [9]. Although it is not a grain, wild rice is recognized as a ‘whole grain’ [10]. Unlike conventional rice, wild rice is usually consumed unprocessed, meaning that wild rice maintains its natural outer layers and contains significantly higher amounts of dietary fiber, micronutrients, and phytochemical compounds. Another important difference between wild rice and conventional white rice is the type of starch they produce [9]. Wild rice contains resistant starch, being often considered to act like a prebiotic; prebiotics are compounds within foods that beneficially affect gut bacterial population and diversity [11]. Gut bacteria produce many metabolites that can either benefit or harm the cardiovascular system [12].

We have previously reported cholesterol-lowering effects and anti-atherosclerotic properties of plant sterols in apolipoprotein E knockout (apo E-KO) mice [13,14]. Over the past few years, we also tested the potential anti-atherosclerotic effects of wild rice in LDL receptor knockout (LDL-r-KO) mice [15,16]. In these studies, we observed significant anti-atherogenic effects of wild rice; however, we were not able to identify a mechanism of action. Atherosclerosis is a multi-factorial disease, in which alterations in inflammatory pathways and oxidative stress, including LDL particle oxidation, play a major role [17]. Furthermore, recent studies reported an association between gut microbiome biology and atherogenesis [18]. Therefore, this study aimed to investigate the impact of wild rice on bacterial species abundance and diversity from 16S rDNA data analysis collected from mouse feces and monitor the metabolic products from the feces and plasma of LDL-r-KO mice.

## 2. Materials and Methods

### 2.1. Animals and Diets

Sixteen male, four week old LDL-r-KO mice were purchased from the Jackson Laboratory, USA. The animals were kept in pairs while using standard cages and fed regular mouse chow in a controlled environment for one week. After a week of chow adaptation, fasting blood samples were taken from the jugular vein under light anesthesia; body weight was also recorded. Plasma total cholesterol was estimated, and the animals were divided into two groups of treated (*n* = 8) and controls (*n* = 8), as previously reported [15]. The treated group was fed an atherogenic diet that contained 60% (*w*/*w*) wild rice powder, whereas the control group received the same atherogenic diet without wild rice powder, as previously reported [15]. Briefly, the mouse chow diet contained 9% fat that was purchased from Ren’s Feed & Supplies Ltd. (Whitby, ON, Canada). This diet was supplemented with 0.06% (*w*/*w*) cholesterol to make it atherogenic; the atherogenic diet was further supplemented with or without 60% (*w*/*w*) wild rice powder and then used for this study. This supplementation was performed by replacing the atherogenic diet by the ground wild rice at 60%. Therefore, the amounts and types of dietary fiber in the control diet and the wild rice diet were not identical. The experiments lasted for 24 weeks.

### 2.2. Sample Collection

The blood samples were taken every four weeks. Fecal samples were collected and stored at −80°C until analysis. At autopsy, final blood samples were taken from the hearts and animals were euthanized while using CO_2_ gas followed by cardiac puncture [15]. The hearts and aortae were collected for the assessment of atherosclerotic lesion development [15]. The Animal Care Committee approved the study at the University of Manitoba, Winnipeg, Canada; refer to Protocol number 18-048 [15].

### 2.3. Plasma Cytokine Levels

Plasma samples that were taken at week 16 of the experiments were used for the estimation of 35 inflammatory biomarkers, using Meso Scale Discovery U-PLEX multiplex assay kit for a mouse (Meso Scale Diagnostics, Rockville, MD 20850-3173, USA) [19]. These markers include interleukins (IL-2, IL-4, IL-9, IL-10, IL-13, IL-17A, IL-17E/IL 25, IL-17F, IL-21, IL-22), tumor necrosis factor-alpha (TNF-α)), TH1/TH2 Combo (IL-1β, IL-5, and IL-12p70A), TH17 Combo 1 (IL-17C, IL-23, and IL-33),TH17 Combo 2 (IL-6, erythropoietin (EPO), IL-27p28/IL-30, vascular endothelial growth factor A (VEGF-A), IL-15, IL-16, and IL-17A/F), interferon gamma-induced protein-10 (IP-10), growth regulated oncogenes (KC/GRO), monocyte chemo-attractant protein-1 (MCP-1), macrophage inflammatory proteins (MIP-1α, MIP-1β, MIP 2, and MIP-3α), granulocyte-macrophage colony-stimulating factor (GM-CSF), and interferon-gamma (IFN-γ). This cytokine analysis was performed on the pooled samples (*n* = 4). MSD SI2400 Imager device and MSD Workbench 3.0 software were used to detect and analyze the standard curves and intensity of the cytokines. The intensity for each biomarker was included in statistical analysis and then reported herein.

### 2.4. Fecal Microbiome Analysis

Microbial diversity and species changes in mice that were fed wild rice as compared to controls were estimated based on extracted 16S rDNA from fecal samples that were collected from pairs of mice at weeks, 0, 4, 16, and 24 during the study. Feces from four cages, where each cage contained two mice (eight mice total), were collected (*n* = 4) for each experimental diet group and stored at −80 °C. Microbial genomic DNA from each thawed fecal sample were extracted with a QIAamp Fast DNA Stool Mini kit (51604, QIAGEN Inc., Germantown, MD, USA), according to its recommended DNA extraction procedures. Fecal DNA was resuspended in nuclease-free water, where the DNA quantity and quality were assessed while using a Qubit™ dsDNA BR Assay Kit (Q32853, Life Technologies, Carlsbad, CA, USA). Fecal DNA samples were stored at −20 °C until they were shipped on dry ice to LC Sciences, LLC (Houston, TX, USA) for 16S rDNA sequencing services. The sequencing methodology that was used by this service and for this study was described previously [20]. Briefly, 16S rDNA sequencing with an Illumina MiSeq platform was carried out, using 338F/806R primers. Further amplification of V3 and V4 regions (around 469 bp in length) was performed by the polymerase chain reaction (PCR). Bioinformatics analysis of 16S rDNA sequence data was assisted by LC Sciences LLC (Houston, TX, USA). Briefly, QIIME software 1.9.1 was used to analyze paired-end reads that were merged into single tags, according to the overlapped region between pairs. The tags were filtered based on their Phred quality score (Q20 and Q30). Chimera sequences that were generated during PCR amplification of the 16S rDNA gene were also excluded, resulting in the final dataset for analysis. This 16S rDNA sequences in the dataset were mapped to the ribosome database project (RDP; http://rdp.cme.msu.edu/) and NCBI 16S rDNA Microbial databases (NT-16S; ftp://ftp.ncbi.nlm.nih.gov/blast/db/nt.gz; as of August 2018) to produce taxonomically annotated sequences, which are referred to as operational taxonomic units (OTUs), described herein. The sequence dataset was grouped using the UCLUST algorithm program. A minimum sequence identity of 99% was used to align the most abundant sequences within each OTU against the reference database sequences, and the hypervariable regions were removed and used to classify the OTUs.

### 2.5. Metabolomics Studies

Metabolites from fecal and plasma samples from week 18 of the study were analyzed by a previously described the liquid chromatography (LC)-mass spectrometry (MS/MS) analysis method [21]. This method combines derivatization and extraction of analytes from the samples, and the selective mass-spectrometric detection using multiple reaction monitoring pairs. The isotope-labeled internal standards were used for metabolite quantification. A total of 133 metabolites were included in the full panel. This analysis was performed through a service contract with The Metabolomics Innovation Centre (TMIC) at the University of Alberta, Edmonton, Canada. It is acknowledged that the use of fecal samples from two mice that were housed in one cage is a limitation for microbiome studies as each mouse can behave as a single ecosystem; however, the average changes among multiple mice were the objective of this study.

### 2.6. Atherosclerotic Lesion Assessment

Sections from the beginning of the aortae were cut and processed for morphological evaluation of the atherosclerotic lesions, as previously described [15]. The sections were stained with hematoxylin and eosin (H&E) and trichrome. Light microscopy techniques were used for semi-quantitative analysis of atherosclerotic lesions in the wild rice treated and control mice [15].

### 2.7. Statistical Analysis

Non-parametric Mann–Whitney tests (also known as the Wilcoxon rank-sum test) and Kruskal–Wallis rank-sum tests were used to calculate the *p*-values and identify significant differences between the two groups of wild rice fed and control mice with an *n* = 4. These statistical analyses were also used to identify significant differences between time course measurements for each animal group when appropriate. Statistical analyses of fecal microbial composition differences were assessed by non-parametric tests, as described by White et al. 2009 [22]. The Venn diagrams of OTUs determined from these analyses were generated while using ‘R’ statistics software (version 3.6.1, https://www.r-project.org/) ‘Venn Diagram’ package to show the number of common OTUs in feces of control and wild rice diet groups. Data are presented as means and standard deviations, where *p*-values ≤ 0.05 were deemed to be significantly different based on the degrees of freedom for each sample group. All of the statistical analyses were performed, while using either Microsoft Office Excel (365, Microsoft, Redmond, USA) or the comprehensive ‘R’ Archive Network (CRAN) statistics software (version 3.6.1, https://www.r-project.org/), with the ‘PMCMR’ analysis package, using ‘kruskal.test’ and ‘wilcox.test’ functions.

## 3. Results

### 3.1. Consumption of Wild Rice Was Associated with Changes in Fecal Bacterial Species Populations

**Figure 1:**
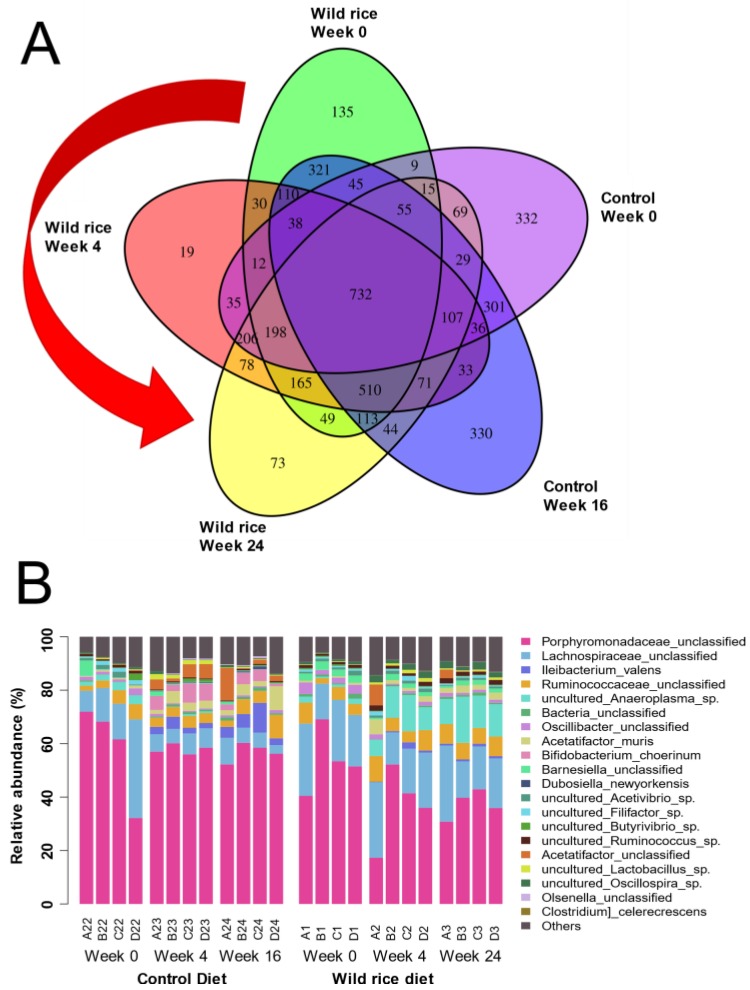
(**A**) Venn diagram comparing 16S rDNA operational taxonomic units (OTUs) from the feces of wild rice and control diet fed mice at weeks 0, 4, 16, and 24. The red curved arrow highlights the decrease in the unique total OTUs in the wild rice fed mice from week 0 to week 24 of the study. (**B**) The relative abundance of the top 20 most abundant bacterial OTUs identified from wild rice or control diet fecal samples at weeks 0 to 24. Identified OTUs are listed according to their order (bottom to top) and color within the bar chart.

Insights into microbial taxonomic alterations could only be confidently determined for high abundance OTUs due to the small number of fecal samples (*n* = 4) examined in this analysis. Microbial 16S rDNA analysis identified more than 200,000 bacterial species (OTUs) in the mouse fecal samples. Figure 1A shows a Venn diagram comparing similar OTUs that were observed between the control and wild rice fed fecal samples collected at various weeks 0, 4, 16, and 24. The majority of all OTUs (732 total) shown in the center of the Venn diagram were identical among all diet treatments, as would be expected in a study involving similar mouse breeds and housing conditions. The wild rice diet fecal samples showed a decrease in the number of unique OTUs over time, where 135 unique OTUs at week 0 reduced to 73 OTUs by week 24. The control samples showed no differences in unique OTUs over time, suggesting that the introduction of the wild rice diet reduced species diversity as compared to the control diets.

Microbial composition changes were further investigated by performing 16S rDNA sequence clustering, where the top 20 most abundant OTUs determined from each fecal sample are shown as a stacked bar chart (Figure 1B). No significant differences were detected between the two major taxa, unclassified Porphyromonadales and Lachnospirales, over time (weeks 0–16 or weeks 0–24) between the control and wild rice diet fecal samples (Figure 1B). Wild rice diets significantly increased (*p* < 0.05) the proportion and appearance of a number of major OTUs when comparing week 0 to week 24 fecal samples; specifically, uncultured *Anaeroplasma sp*. (8.8-fold increase), *Acetatifactor muris* (4.4-fold increase), uncultured *Lactobacillus* sp. (3-fold increase), uncultured *Oscillospira* sp. (3-fold increase)*,* and *Dubosiella newyorkensis* (0.07% appearance) increased (Figure 1B). Losses or significant reductions (*p* < 0.05) in OTUs within the fecal wild rice diet microbiomes after comparing them to the control diet microbiomes were also noted over time (weeks 4 and/or 24). Specifically, reductions in unclassified *Barnesiella* sp. (2-fold reduction), uncultured *Butyrivibrio* sp. (2-fold reduction), and unclassified *Oscillibacter* sp. (2-fold reduction;) were detected. *Bifidobacterium choerinium* was also undetectable in the wild rice diet samples at weeks 4 and 24 as compared to control diet (Figure 1B). Altered proportions of OTUs were also noted within the control diet fecal samples over time (weeks 0 to 16); significant (*p* < 0.05) reductions in uncultured *Anaeroplasma* sp. (undetectable at week 16), uncultured *Ruminococcus* sp. (2-fold reduction), and uncultured *Filifactor* sp. (undetectable at week 16) were noted, as well as significant increases (*p* < 0.05)in *Ileibacterium valens* (2–5% appearance) and *Bifidobacterium choerinum* (43-fold increase). It is noteworthy that the control diet OTUs, as mentioned above, were either low or completely absent in the wild rice diet fecal samples (Figure 1B). Overall, fecal microbiome analyses indicate that the wild rice diet significantly alters many high abundance bacterial species.

**Figure 2:**
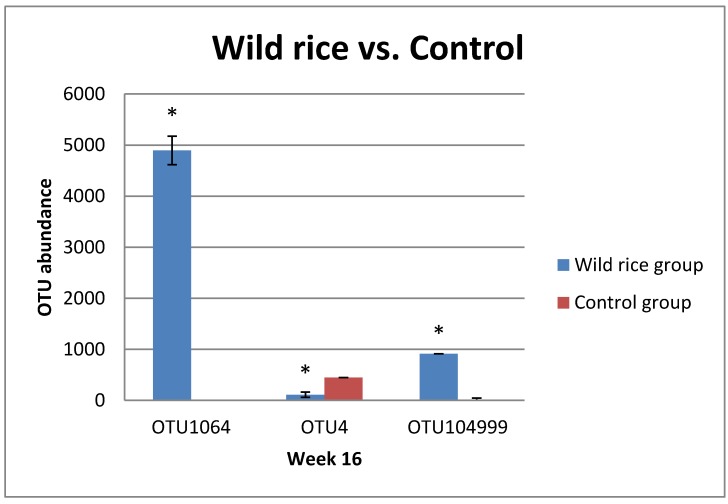
The abundance of OTUs for selected species identified from 16S rDNA analysis of wild rice fed and control fecal samples. All samples were collected at week 16 of the study with an *n* = 4 per group. OTU 4: *Acetatifactor sp.* unclassified; OTU 1064: *Porphyromonadaceae sp.* unclassified; OTU 104999: uncultured *Anaeroplasma sp.* *: *p* < 0.05 as compared with the controls.

Figure 2 shows values for three OTUs that reached statistically significant differences (*p* < 0.05) between the treated and control animals. The abundance of unclassified *Prophyromonadaceae sp*. and uncultured *Anaeroplasma sp*. in wild rice fed mice were approximately 5000 and 1000, respectively, more than those in the control group.

### 3.2. Wild Rice Consumption Is Associated with Changes in Plasma Inflammatory Markers

Our analysis included an estimation of 35 different markers in inflammatory pathways. Statistical analyses between data from the wild rice fed and control groups only identified five markers with a significant change in their mean values (Table 1). The levels of EPO and interleukin 10 (IL-10) increased by approximately 109% and 130%, respectively, in the wild rice diet mice. In contrast, wild rice diet mice had reduced markers of approximately 18%, 18%, and 35% of tumor necrosis factor-alpha (TNF-α), vascular endothelial growth factor (VEGF), and interleukin-16 (IL-16), respectively, as compared with the control animals.

### 3.3. Wild Rice Diets Show Differences in Fecal and Plasma Metabolites

LC-MS/MS analysis of metabolites that were extracted from wild rice and control diet fecal and plasma samples identified a total of 133 metabolites. We performed a Mann-Whitney rank-sum test on metabolite values between the sample groups to improve the confidence in metabolite analyses due to our lower sampling numbers (*n* = 4). We focused our results on metabolites with significant differences from the control diet group (*p* < 0.05). Table 2 show significant changes in the levels of 11 plasma metabolites that were differentially detected. Glucose increased in wild rice fed mice by approximately 61%, whereas 10 metabolites, including short-chain fatty acids (C8, C10, and C12), medium-chain fatty acids (C14:1, and C16), and long-chain fatty acids (C18 and C18:1) decreased by 17–48% in the wild rice diet plasma samples as compared to those in the controls.

Among 24 fecal metabolites listed in Table 3, only four metabolites, butyric acid and three phospholipids increased by 51–323%. The remaining metabolites, which included amino acids, short-chain fatty acids (except butyric acid), and long-chain fatty acids, showed a decrease of 30–70% by wild rice fed fecal samples as compared to those in the control group (Table 3).

### 3.4. Wild Rice Consumption Prevents Atherogenesis

**Figure 3:**
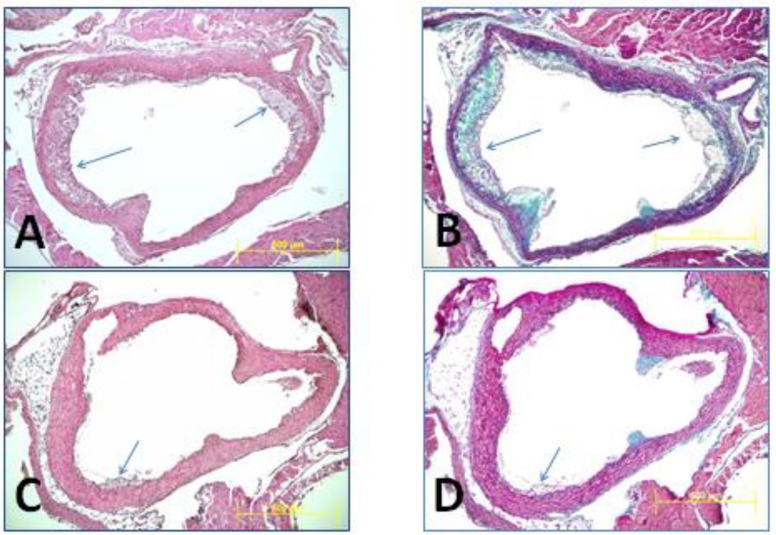
Representative photomicrographs were taken at the beginning of aorta from one control mouse (**A**,**B**) and one wild rice fed mouse (**C**,**D**) illustrating atherosclerotic lesions (arrows). As it is seen in (**A**,**B**), atherosclerotic lesions are large and well established in the control mouse (arrows), while such advanced lesions are missing in the wild rice fed mouse (**C**,**D**). H&E staining (**A**,**C**); trichrome staining (**B**,**D**).

In agreement with our previous findings [15,16], we report that the mice fed with wild rice had much smaller atherosclerotic lesions in their aortae as compared to that in the control animals. Figure 3 illustrates advanced atherosclerotic lesions at the beginning of aortae in the control animals (arrows), but similar lesions were absent or minimal in the similar anatomical region of aortae of the wild rice fed mice.

## 4. Discussion

We have previously shown that wild rice consumption is associated with the prevention of atherosclerotic vascular disease in LDL-r-KO mice [15,16]. This effect could be related to reductions in plasma cholesterol levels. We have shown alterations in LDL-r-KO mice microbiomes may influence the detection of inflammatory markers, and alter concentrations of metabolites when fed a diet rich in wild rice based on the results of our study. LDL-r-KO mice exhibit atherosclerosis, which is known to be an inflammatory disease [23]. Therefore, treatment with agents that possess pro-inflammatory properties are expected to increase the risk for this disease and anti-inflammatory states should prevent atherosclerosis [24]. LDL-r-KO mice fed a wild rice diet had approximately 75% lower atherosclerotic lesions (0.46 ± 0.11 vs. 1.95 ± 0.16 mm^2^) in their aortic roots as compared to the control diet mice [15].

The results from the current study have identified that wild rice feeding is associated with a 130% change increase in IL-10; IL-10 was shown in previous studies to possess anti-atherogenic activities [25]. Another interesting observation was a 109% increase in the levels of EPO in plasma of wild rice fed mice. Recent studies have shown the anti-atherogenic properties for EPO [26]. The mechanism by which IL-10 and EPO levels were increased in wild rice treated animals is not presently understood, but it may be associated with changes in gut microbiome composition. We have reported a beneficial change in the inflammatory pathways of mice that were fed either wild rice or Saskatoon berries [27,28]. Additionally, a recent study monitoring dietary changes in mice demonstrated that specific microbes can alter gut T-cell responses [29]. It is possible that the changes in cytokine concentrations that we observed in wild rice fed LDL-r-KO mice may indirectly influence plasma when phytochemicals produced by altered gut microbiome species reach the blood.

We also reported that starch from wild rice is different in nature from the starch found in conventional white rice; wild rice also contains a significant amount of dietary fiber [9,16]. These forms of carbohydrates may act as prebiotics, which thereby alters the diversity and population of the gut microbiome [11,12]. In the present study, we observed that unclassified Prophyromonadaceae decreased in the fecal samples of wild rice fed mice (Figure 1B). When we examined different OTUs associated with unclassified Prophyromonadaceae, we observed that many OTUs increased in wild rice group as compared to those in the control (Figure 2). This suggests that specific Prophyromonadaceae, such as unclassified *Barnesiella* sp., differ between control and wild rice fed mice (Figure 1B). Previous studies examining changes in mouse gut microbial species showed that mice that were fed with polysaccharides from the mushroom *Auricularia auricular* altered quantities of Prophyromonadaceae in their intestine as compared to control diet animals [30]. This study highlights the importance of carbohydrates on microbial species diversity. In the same mushroom study, the treated animals showed higher serum IgA and IgG, indicating changes in gut microbiome due to mushroom carbohydrate consumption also modulated the immune system of the mice [30]. Another noteworthy observation was the difference in *Anaeroplasma* sp. between the wild rice fed and control fecal samples. A study by Zeng et al. [31] reported an increased abundance of *Anaeroplasma* species in the intestines of wild type mice that were fed a high-fat diet. These authors concluded that high-fat diets promoted colonic aberrant crypt formation accompanied by an increase in the abundance of opportunistic pathogens, such as *Anaeroplasma* sp. in the colon of C57BL/6 mice [31]. *Acetatifactor* sp. was also identified in high abundance over time within the wild rice fed fecal samples. Although not much is known about *Acetatifactor* species’ influence on murine microbiomes, Pfeiffer et al. [32] suggested the name *Acetatifactor muris* due to its isolation from the cecum of mice fed a high-fat diet, which we also observed in our study only among control diet LDL-r-KO mice (Figure 1B). *Acetatifactor* species are not known to metabolize glucose and they are associated with higher phenylalanine arylamidase activities. In our study, we identified that wild rice diets reduced the *Acetatifactor muris* levels, suggesting that this species, might indirectly influence atherosclerosis in an LDL-r-KO mouse model.

Analysis of fecal metabolic compounds revealed that wild rice consumption was associated with altered metabolite abundances, particularly metabolites that are associated with amino acids, carbohydrates, and fats. All amino acids that were detected in fecal samples were significantly reduced from mice fed wild rice diets. Wild rice diets may promote the growth and predominance of these amino acid utilizing species to catabolize more amino acids, since the most abundant bacterial species *Anaeroplasma sp*., *Acetatifactor sp*., and *Prophyromonadaceae sp*. significantly differed in wild rice as compared to the control group. For example, *Acetatifactor muris* may be a species promoting greater amino acid usage, as it possesses phenylalanine arylamidase, which breaks down L-phenylalanine from peptides [32]. This might suggest that a significant reduction in the concentrations of several amino acids could be associated with bacteria containing this and other relevant enzymes.

Among several short-chain fatty acids and their derivatives, butyric acid was found in the fecal materials from the wild rice fed mice 81% more than that in the control animals. This finding coincides with specific and significant increases in butyric acid-producing uncultured *Butyrivibrio* species identified from 16S rDNA wild rice fed fecal microbiomes (Figure 1B). *Butyrivibrio* sp. is commonly enriched in the guts of ruminant animals, where they produce butyrate from the breakdown of plant fibers and structural carbohydrates, specifically hemicellulose [33,34]. Many studies have reported the metabolic benefits of short-chain fatty acids [35,36]. Analysis of blood plasma samples did not correlate well to metabolic and microbiome changes despite increases in butyrate in the intestine of wild rice fed mice. Plasma concentrations of other short-chain fatty acids, such as caproic acid and caprylic acid, were significantly lower in the wild rice fed mice as compared to those in the controls. However, there was an association between the levels of long-chain fatty acids in the fecal and plasma samples, as these levels were increased in both samples from the wild rice fed mice as compared to those in the controls. Another observation was a significant increase in plasma glucose concentrations in the wild rice fed mice as compared to that in the control group. Increased plasma glucose levels are seen during diabetes or insulin resistance in animals as well as humans [37,38]. However, the consumption of high fiber diets is generally recommended to combat complications that are caused by diabetes [39,40]. Wild rice is a rich source of dietary fiber; therefore, this observation seems to be in contrast with our general knowledge and it certainly begs more investigation. To conclude, additional investigations examining the interplay between changes in intestinal microflora, inflammatory response, and metabolic biomarkers are warranted, as they may play a role in the pathogenesis of chronic diseases, like atherosclerosis. Overall, it should be mentioned that a low number of animals, pooled fecal, and plasma samples, as well as a lack of different doses of wild rice, could be viewed as limitations of the present study.

## 5. Conclusions

In conclusion, we hereby report that the long term consumption of wild rice at 60% (*w*/*w*) in LDL-r-KO mice is associated with the prevention of atherosclerosis. This effect was accompanied by significant alterations in the fecal bacterial population and diversity, as well as significant changes in several inflammatory and metabolic biomarkers. Of particular interest was an increase in the plasma glucose levels in the wild rice fed mice; currently, we have no explanation for this finding. Other findings that can support anti-atherogenic properties of wild rice are increases in the plasma levels of anti-inflammatory marker IL-10 and EPO. Altogether, this study provides preliminary evidence in support of additional studies on this animal model and others to improve our understanding of how gut bacterial species, plasma inflammatory markers, and metabolic biomarkers may prevent atherosclerosis. Furthermore, a dose-response study can help to establish whether lower doses of wild rice can result in similar findings in this animal model.

## Figures and Tables

**Table 1:** Plasma cytokine intensity from the wild rice fed and control groups.

Plasma Cytokines (pg/mL)	Experimental Groups		
	Control Group (*n* = 4)	Wild Rice Group (*n* = 4)	% Change
EPO	6.69 ± 2.7	14.01 ± 4.7 *	↑109
TNF-α	6.77 ± 0.7	5.57 ± 0.5 *	↓18
VEGF	6.06 ± 0.6	5.0 ± 0.5 *	↓18
IL10	4.32 ± 1.77	9.94 ± 3.14 *	↑130
IL16	645.83 ± 14.4	422.07 ± 64.3 *	↓35

Data are presented as means ± standard deviation. Statistical analyses were performed using the Mann Whitney test; *: *p* < 0.05 as compared with the controls. EPO: erythropoietin, TNF-α: tumor necrosis factor-α, VEGF: vascular endothelial growth factor, IL-16: interleukin-16. ↓: Decrease. ↑: Increase

**Table 2:** Metabolomics data from plasma samples of mice fed wild rice and control diets.

Plasma Metabolomics		Control Group (*n* = 4) (µM)	Wild Rice Group (µM) (*n* = 4)	% Change from Control Diet
Nutrients	Metabolites			
Proteins	Putrescine	1.21 ± 0.19	0.83 ± 0.16 *	↓32%
Carbohydrates	Glucose	10,208.17 ± 2575.4	16,405.98 ± 2966.73 *	↑61%
Short Chain Fatty Acids	Caprylic acid	0.06 ± 0.009	0.05 ± 0.007 *	↓25%
	Capric acid	0.09 ± 0.009	0.06 ± 0.010 *	↓30%
	Lauric acid	0.08 ± 0.014	0.05 ± 0.0002 *	↓41%
Medium Chain Fatty Acids	Myristic acid (C14)	0.17 ± 0.026	0.10 ± 0.008 *	↓41%
	3-Hydroxytetradecenoyl-carnitine (C14:1-OH)	0.03 ± 0.0047	0.02±0.0027 *	↓29%
	Palmitic acid (C:16)	0.51 ± 0.158	0.32 ± 0.032 *	↓38%
	Hydroxyhexadecadienyl-l-Dcarnitine (C16:2OH)	0.01 ± 0.0026	0.01 ± 0.002 *	↓35%
Long Chain Fatty Acids	Stearic (C18)	0.18 ± 0.0205	0.12 ± 0.012 *	↓31%
	Hydroxy-Oleyl-l-Carnitine (C18:1OH)	0.05 ± 0.011	0.03 ± 0.002 *	↓34%

Data are presented as means ± standard deviation. Statistical analyses were performed using the Mann-Whitney test; *, *p* < 0.05 as compared with controls. ↓: Decrease. ↑: Increase

**Table 3:** Fecal metabolomics data from the wild rice fed and control groups.

Fecal Metabolomics Assay		Control Group (*n* = 4)	Wild Rice Group (*n* = 4)	% Change
Nutrients	Metabolites (µM)			
Amino acids	Glycine	1.15 ± 0.529	0.34 ± 0.12 *	↓70%
	Alanine	2.79 ± 1.31	1.18 ± 0.32 *	↓58%
	Proline	0.52 ± 0.179	0.18 ± 0.07 *	↓66%
	Valine	0.81 ± 0.381	0.26 ± 0.13 *	↓67%
	Leucine	0.92 ± 0.497	0.32 ± 0.11 *	↓66%
	Isoleucine	0.91 ± 0.424	0.28 ± 0.11 *	↓69%
	Methionine-sulfoxide	0.16 ± 0.061	0.06 ± 0.04 *	↓60%
	Tryptophan	0.07 ± 0.034	0.03 ± 0.01 *	↓62%
Short-Chain Fatty acids	Butyric acid	0.11 ± 0.029	0.20 ± 0.05 *	↑81%
	Succinic acid	0.18 ± 0.088	0.08 ± 0.01 *	↓58%
	Isobutyric acid	0.07 ± 0.014	0.03 ± 0.01 *	↓60%
	Methylmalonic acid	0.003 ± 0.0003	0.0009 ± 0.004 *	↓72%
	Dodecanedioyl-l-Carnitine (C12DC)	7.93 × 10^−5^ ± 8.72 × 10^−6^	4.44 × 10^−5^ ± 9.71 × 10^−6^ *	↓44%
Long-Chain Fatty Acids	Vaccenic acid (C18:1)	3.50 × 10^−5^ ± 1.33 × 10^−5^	1.63 × 10^−5^ ± 1.40 × 10^−6^ *	↓53%
	Lenoleic acid (C18:2)	6.01 × 10^−5^ ± 8.50 × 10^−6^	3.48 × 10^−5^ ± 3.21 × 10^−6^ *	↓42%
Phospholipids	LYSOC16:1	0.0002 ± 4.98 × 10^−5^	0.0004 ± 9.77 × 10^−5^ *	↑72%
	LYSOC16:0	0.005 ± 0.0009	0.01 ± 0.002 *	↑93%
	LYSOC18:2	0.001 ± 0.0003	0.002 ± 0.001 *	↑157%
	LYSOC18:1	0.001 ± 0.0002	0.004 ± 0.001 *	↑226%
	16:1SM	0.0001 ± 3.57 × 10^−5^	4.91 × 10^−5^ ± 5.62 × 10^−6^*	↓69%
	20:2SM	1.43 × 10^−5^ ± 5.50 × 10^−7^	2.15 × 10^−5^± 5.64 × 10^−6^ *	↑51%
	PC36:6AA	8.01 × 10^−5^± 3.10 × 10^−5^	0.0003 ± 8.66 × 10^−5^ *	↑323%
	PC38:0AA	0.0004 ± 9.23 × 10^−5^	0.0003 ± 3.83 × 10^−5^ *	↓30%
	PC40:2AA	6.93 × 10^−5^ ± 1.39 × 10^−5^	4.33 × 10^−5^ ± 2.19 × 10^−6^*	↓37%

Data are presented as means ± standard deviation; each fecal sample represents feces from 2 caged mice, eight mice total and *n = 4 fecal* samples per experimental group. Statistical analyses were performed using the Kruskal Wallis test; *, *p* < 0.05 as compared to the controls. ↓: Decrease. ↑: Increase
